# Practical structure solution with **ARCIMBOLDO**

**DOI:** 10.1107/S0907444911056071

**Published:** 2012-03-16

**Authors:** Dayté Rodríguez, Massimo Sammito, Kathrin Meindl, Iñaki M. de Ilarduya, Marianus Potratz, George M. Sheldrick, Isabel Usón

**Affiliations:** a Instituto de Biología Molecular de Barcelona (IBMB–CSIC), Barcelona Science Park, Baldiri Reixach 15, 08028 Barcelona, Spain; b Lehrstuhl für Strukturchemie, Universität Göttingen, Tammannstrasse 4, 37077 Göttingen, Germany; c Institució Catalana de Recerca i Estudis Avançats (ICREA), Spain

**Keywords:** *ARCIMBOLDO*, fragment search, *Phaser*, density modification, multi-solution phasing, *SHELXE*

## Abstract

Since its release in September 2009, the structure-solution program *ARCIMBOLDO*, based on the combination of locating small model fragments such as polyalanine α-helices with density modification with the program *SHELXE* in a multisolution frame, has evolved to incorporate other sources of stereochemical or experimental information. Fragments that are more sophisticated than the ubiquitous main-chain α-­helix can be proposed by modelling side chains onto the main chain or extracted from low-homology models, as locally their structure may be similar enough to the unknown one even if the conventional molecular-replacement approach has been unsuccessful. In such cases, the program may test a set of alternative models in parallel against a specified figure of merit and proceed with the selected one(s). Experimental information can be incorporated in three ways: searching within *ARCIMBOLDO* for an anomalous fragment against anomalous differences or MAD data or finding model fragments when an anomalous substructure has been determined with another program such as *SHELXD* or is subsequently located in the anomalous Fourier map calculated from the partial fragment phases. Both sources of information may be combined in the expansion process. In all these cases the key is to control the workflow to maximize the chances of success whilst avoiding the creation of an intractable number of parallel processes. A GUI has been implemented to aid the setup of suitable strategies within the various typical scenarios. In the present work, the practical application of *ARCIMBOLDO* within each of these scenarios is described through the distributed test cases.

## Abstract 2

**ARCIMBOLDO** combines the location of small fragments with *Phaser* and density modification with *SHELXE* of all possible *Phaser* solutions. Its uses are explained and illustrated through practical test cases.

## 1. Introduction

Dual-space recycling *ab initio* methods for phasing equal-atom macromolecular structures that assume atomicity require atomic resolution data (Miller *et al.*, 1993 ▶; Sheldrick *et al.*, 2001 ▶). To overcome this barrier and push the limit to lower resolution, additional information or alternative constraints are required. Exploiting the presence of heavy atoms in the structure (Caliandro *et al.*, 2008 ▶) or extrapolating unmeasured reflections up to atomic resolution (Caliandro *et al.*, 2005*a* ▶,*b* ▶; Jia-xing *et al.*, 2005 ▶) have proven to be useful. Alternatively, the fact that macromolecules are made up from building blocks of known geometry that can be predicted from their amino-acid sequence, such as α-helices, can be enforced as an alternative to atomicity as a means of bringing in prior stereochemical information. One of the problems atomic resolution *ab initio* methods suffer from at lower resolution is that the figures of merit are no longer reliable. Indeed, the *E*-based correlation coefficient (Fujinaga & Read, 1987 ▶) of partial solutions is invariably high for the expected number of atoms. In a multisolution frame, there is no use in producing correct solutions if they cannot be discriminated from among the rest, as a manual check of all solutions would not be practicable. Fragment location in combination with density modification has enabled the solution of previously unknown protein structures at resolutions of up to 2 Å and the identification of the correct phases from the figures of merit characterizing the partial main-chain trace of the resulting map through its CC and number of residues traced. This method has been implemented in the program *ARCIMBOLDO* (Rodríguez *et al.*, 2009 ▶), which combines multisolution location of small (10–14 amino acids) extremely accurate models such as polyalanine α-helices with the program *Phaser* (McCoy *et al.*, 2007 ▶) and density modification and autotracing with the program *SHELXE* (Sheldrick, 2008 ▶). Academics can download *ARCIMBOLDO* from the web (http://chango.ibmb.csic.es/ARCIMBOLDO) for free.

The program is named after the Italian painter Giuseppe Arcimboldo (1527–1593), who assembled portraits from fruit and vegetables. Analogously, the method tests many hypotheses assembled from secondary-structure fragments and, while most of them remain a ‘still life’, density modification is effective in revealing and identifying the true portrait of the protein being solved.

Since its first use for the *ab initio* case, *ARCIMBOLDO* has been expanded to other scenarios that enable it to tackle larger structures with poorer data, and its user friendliness has been improved through the incorporation of a GUI to help the user set up and parameterize an *ARCIMBOLDO* run. The same frame as that used for the *ab initio* case allows the exploitation of other sources of previous stereochemical knowledge, such as low-homology models or experimental phases from derivatives that are too noisy to be interpretable on their own. The present work describes the use of *ARCIMBOLDO* in its different modes and discusses its application to various test data sets.

## 2. Requirements for the computer system, the Condor grid

*ARCIMBOLDO* is very demanding in terms of computational power. Thus, it is coupled to the use of a grid running Condor (Tannenbaum *et al.*, 2002 ▶), where the many jobs that have to be run can be calculated in parallel. The procedure is intrinsically straightforward to parallelize: the available pool of machines can be used to distribute the various jobs, such as each rotation search, each translation search, packing filtering or refinement of a fraction of the solutions, or individual density-modification runs on selected partial structures. Solutions are aggregated and output evaluated to control the overall flow to optimize resources. The tests described were run on a local grid made up of 100 nodes totalling 175 Gflops and on a remote grid with 248 nodes and 500 Gflops on the supercomputer Calendula from the FCSCL in León, Spain. Extension to other middleware systems is intended in the future.

## 3. *ARCIMBOLDO* uses

**Figure 1:**
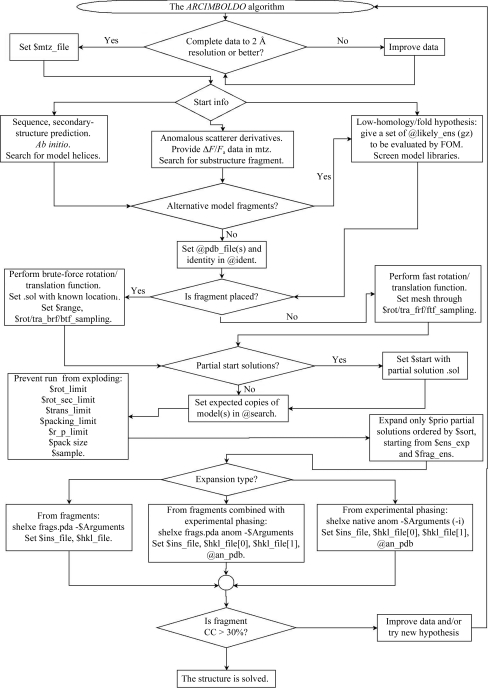
Scheme showing the alternative process flow and variables and files to be set up to run *ARCIMBOLDO*.

The conditions, use scenarios and flow control, together with the necessary files and parameters to be set within *ARCIMBOLDO*, are summarized in the flowchart in Fig. 1 ▶. A preliminary requirement is the availability of complete throughout (ideally, as much as possible in the low-resolution range as in the high-resolution range) good-quality diffraction data to a resolution of 2 Å or better. This is passed to the program through a .mtz file and the labels where F and SIGF are to be found have to be provided to the script. Exceptionally, successes have been experienced with problematic data (non­merohedrally twinned) or at poorer resolution (2.1 Å). Often 2.5 Å resolution data is sufficient for successful fragment location, but as the resolution becomes worse expansion from the partial structure through density modification and autotracing does not succeed. In such cases, a more complete starting hypothesis or additional information will be needed in order to reach a successful solution and prevent the density-modification expansion from becoming stuck.

Structures have been solved in any crystal system or with noncrystallographic symmetry as high as sevenfold; thus, symmetry does not pose an intrinsic limit. The possible size limit on the structures to be tackled by these methods is strongly related to the computational power available: larger structures require the location of more fragments and more trials. Success may be reached by increasing the CPU power. Intrinsic barriers have so far not been determined but cannot be excluded.

### 3.1. *Ab initio*

The simplest case is when all the available previous information is reduced to a set of native data and the amino-acid sequence. From this, secondary-structure prediction algorithms can derive the number and length of expected α-­helices and β-strands. α-Helices tend to be very constant in their main-chain geometry, especially over a short range (10–14) of amino acids. In contrast, the higher variability and shorter span of β-strands make them less useful as search fragments and so far no *ab initio* successes have been obtained exploiting their presence.

Different fragments can be input to *ARCIMBOLDO* and the order and number of copies to be located for each of them has to be specified. When searching for helices of different length, it is convenient to start with the larger ones, as short helices could also be accommodated in the sites of the longer ones and packing filters would prevent further progress.

As the fragments used for *ab initio* are small but very accurate (*i.e.* have a small r.m.s.d. to part of the true structure), a smaller mesh should be selected rather than the default *Phaser* grid. Empirical values found to be suitable are 1.0–3.0° for the rotation search and 0.3–0.6 Å for the translation search.

As an example of *ab initio* phasing, we will consider the structure of PRD2 in space group *P*2_1_ (PDB entry 3gwh; Rodríguez *et al.*, 2009 ▶). The asymmetric unit contains 222 protein residues making up ten helices, with eight of them between 14 and 20 amino acids long. Thus, it is appropriate to search for helices of 14 amino acids. In this case, three such fragments are necessary before expansion of the partial structure succeeds.

**Figure 2:**
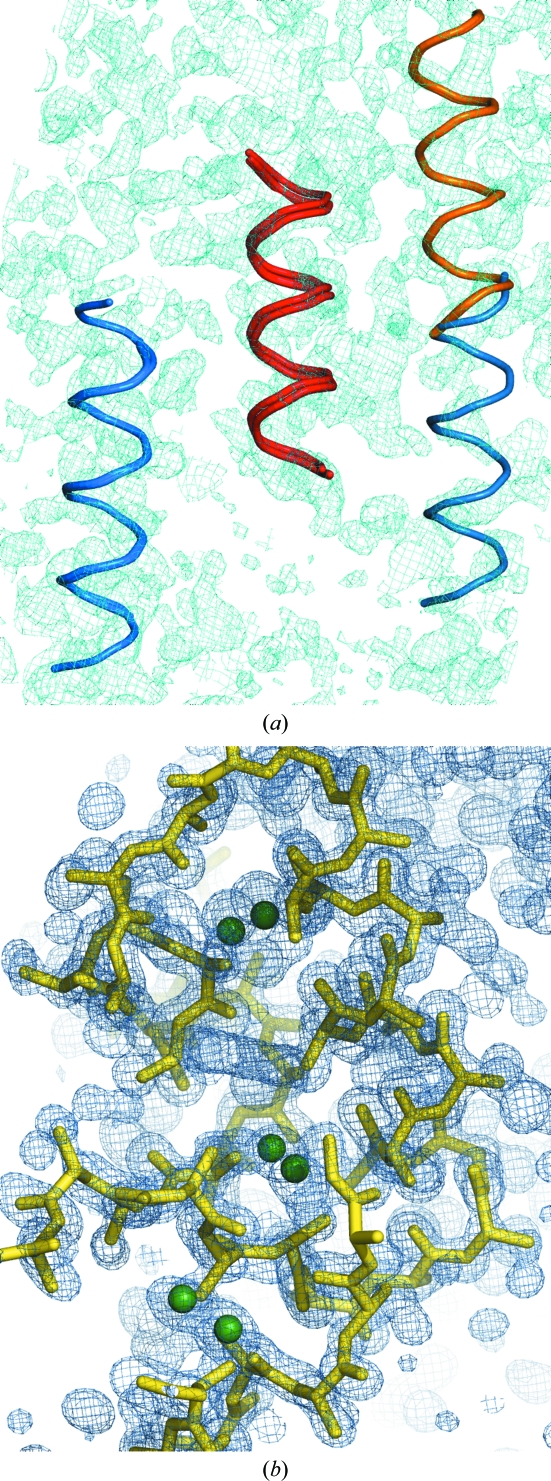
Placed fragments and resulting map or structure for the test cases. (*a*) The three three-fragment substructures leading to structure solution in the case of PRD2 when using model helices of 14 alanines. Whereas the helices depicted in red and orange are common to all three solutions, the blue one is slightly different for two of them and partially overlapped and out of density for the third. The resulting electron-density map after expansion of the best solution is shown in cyan contoured at 1σ and including data extrapolated to 1.7 Å. (*b*) Located anomalous substructure shown as green spheres derived from the S atoms of hellethionin D (NMR structure; PDB entry 1nbl; Milbradt *et al.*, 2003 ▶), the resulting electron-density map after expansion, shown in blue contoured at 1σ and including data extrapolated to 1.0 Å, and the polyalanine trace of the solution obtained for viscotoxin A1 after the first fragment.

The run is set up by cutting the resolution for the fragment search at 2.5 Å, the rotation step at 1.5° and the translation step at 0.7 Å. After every step solutions are limited, if necessary, to keep their number within tractable limits. Furthermore, after each step fragment expansion is attempted on the ten solutions with the highest *Z* score characterizing their translation function. The parameters used for the *SHELXE* expansion are 30 cycles of density modification without sharpening alternating with three rounds of auto­tracing, no sharpening, deriving phases from the fragments to the resolution limit of 1.9 Å and extrapolating missing reflections up to 1.7 Å. In the case of the first and second fragment, none of these solutions showed a mean phase error (MPE) against the final structure better than 87°. For the third fragment, one of the top ten solutions showed an MPE of 57°. Having found a solution within this subset, the remaining ones were not expanded. In this case, expansion of all 153 solutions consisting of three fragments would phase the structure in two more cases. Fig. 2 ▶(*a*) displays the three sets of fragments. The helices in red and orange are common to all three winning solutions and the blue ones are different in each case, although the two on the left overlap over a large span (initial MPE of the fragment phases of 63°) and the one on the right is rather incorrectly placed (initial MPE of 74°). The final map, shown in cyan, reveals that their positions are otherwise extremely accurate.

### 3.2. Anomalous/MAD data

Combination of molecular replacement and weak anomalous data has proved to be useful for automatic structure solution (Panjikar *et al.*, 2009 ▶) or to tackle difficult cases (Lira-Navarrete *et al.*, 2011 ▶). Experimental phase information can be exploited and integrated into the *ARCIMBOLDO* flow in three different ways.

It is possible to search for substructures made up of anomalous scatterers or heavy atoms if a suitable model is known *a priori*. Although this is not a frequent scenario, it could arise, for instance, if the structure to be determined happens to contain a cluster or cofactor with several anomalous scatterers in a known geometry or a fold with a known disulfide-bridge pattern where the coordinates for the S atoms can be taken from a homologous structure.

The .mtz file passed on to *Phaser* must contain the Δ*F* or *F*_A_ data and their standard deviations. These columns are set as F and SIGF in the *ARCIMBOLDO* script.

The viscotoxin A1 (VTA) structure in space group *P*4_3_2_1_2 (PDB entry 3c8p; Pal *et al.*, 2008 ▶) provides a suitable case to illustrate this. Data recorded to 1.25 Å resolution using an in-house Cu *K*α system show significant anomalous signal derived from six cysteines involved in three disulfide bridges present in each of the viscotoxin molecules. A fragment consisting of the six cysteines can be extracted from another PDB entry displaying the viscotoxin fold, such as the NMR structure of hellethionin D (Milbradt *et al.*, 2003 ▶). In the fragment, the remaining atoms of the cysteine residues were retained with occupancy 0. They should not be considered as part of the fragment as they do not present anomalous diffraction, but are still useful to compute a packing filter to discard solutions that must necessarily clash.

Search and optimization with this anomalous fragment was performed cutting the anomalous data to a resolution of 2 Å. The first fragment produces 20 translation solutions, of which five are unique and have similar figures of merit. At the expansion stage, *SHELXE* uses the anomalous substructure and the file containing the anomalous differences and phase shifts to phase the native data, in combination with fragments, if present. In this case, 30 cycles of density modification and three cycles of autotracing already bootstraps with one six-S-­atom substructure. If the second anomalous fragment is searched for, the correct solution is even clearer from the figures of merit (LLG = 180 *versus* 119 and TFZ = 8.6 *versus* 5.2 for the next best). Fig. 2 ▶(*b*) shows the density map and main-chain trace obtained as well as the sulfur substructure.

The second possibility is the combination of experimental phases and fragments. In most cases where anomalous or MAD data are available, the substructure can be determined more effectively by dual-space recycling methods. If the experimental phases derived from the substructure are not accurate enough to provide an interpretable structure solution, they can be input into *ARCIMBOLDO* and combined with the search for model fragments. In this case, it is possible to restrict the search for model fragments and perform brute-force rotation and/or translation searches, as a secondary-structure element linked to an anomalous fragment might be predictable, such as the two helices linked through a disulfide bridge in the VTA fold or in fact any case where a cysteine would be contained in a region predicted to be α-helical. A key point is that substructure and fragments have to refer to the same origin if their phasing information is to be combined. In many space groups refinement allows partial solutions to drift away from the starting position in one or more directions. *ARCIMBOLDO* can be restarted from any point in its flow. This allows the input of any kind of previous information, be it a partial solution made up of fragments, an anomalous substructure or a combination of both. When searching for further fragments, the anomalous fragment must be input as part of the native solution.

A third alternative is deriving an anomalous map to search for the substructure from the phases provided by a partial model. In this case the structure is probably good enough for autotracing to bootstrap, but recycling the search for the substructure is much faster than autotracing, and combining both sources of information probably renders a better final map.

### 3.3. Alternative fragments

Exploiting any particular stereochemical knowledge that may be available is possible. For instance, side chains may be modelled on a predicted helix and various combinations of the most frequent conformers may be set up. Even if no homologous structure leads to a successful molecular-replacement solution, poor homology models will provide a reasonable hypothesis about the general fold, as would particular local knowledge of an active site. In such cases, rather than building up the fold from sequentially added model fragments, it is possible to dismember the model into pieces and input them as search fragments. Usually, such information opens up several possibilities that have to be tested and, ideally, confirmed or discarded early on. *ARCIMBOLDO* provides a means of testing a list of alternative fragments in parallel and specifying a figure of merit (LLG or *Z* score) to let the procedure select the optimal one. This list may be a file explicitly input into the script or passed as an external file containing one PDB or one gzipped tar file of multiple PDB files in each line.

**Figure 3:**
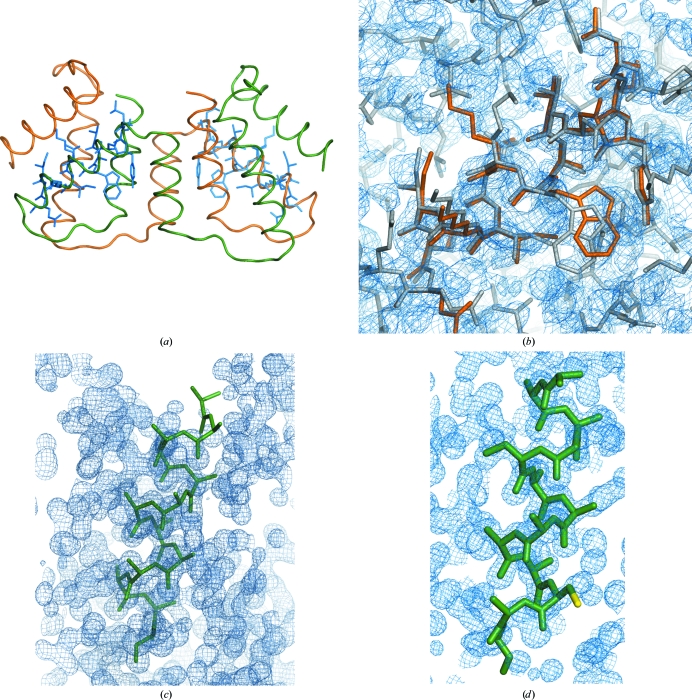
Structure solution with fragments with side chains. (*a*) Final structure of PRD2 shown as a backbone trace, with superimposed helices with side chains modelled in standard conformations, as located in the successful solution. (*b*) Detailed view of one of these helices superimposed on the final structure and the resulting electron-density map after *SHELXE* expansion contoured at 1σ and including data extrapolated to 1.7 Å. (*c*) Slightly misplaced polyalanine helix that nevertheless leads to structure solution in the case of viscotoxin A1. (*d*) Helix with cysteine side chain (yellow) in standard conformation used in the case of viscotoxin A1. In this case part of the helix is also misplaced, including the cysteine side chain, but this substructure also leads to a final electron-density map shown in cyan contoured at 1σ, including data extrapolated to 1.0 Å and characterized by a mean phase error of 18.9°. Figures were prepared with *PyMOL* and *Coot* (DeLano, 2002 ▶; Emsley *et al.*, 2010 ▶).

The same example as provided for the *ab initio* case, PRD2 in space group *P*2_1_ (PDB entry 3gwh), is used here. Four alternative fragments are proposed: a model polyalanine helix with 14 residues; a helix with side chains in the most represented conformers from Leu74 to Gln87 modelled with *SCWRL*4 (Krivov *et al.*, 2009 ▶); the same helix with the side chains in the standard conformers that are closest to the final structure and the real helix cut out from monomer *A* in the final structure but with artificial *B* factors. The figure of merit used to select the fragment was the LLG of the rotation function. After calculating the rotation search for every PDB input with data truncated to 2.1 Å resolution, the figures obtained were 10.0 for the polyalanine helix and 10.1 for the helix with the most frequent conformers, while the real helix and that with the closest conformers both scored 11.4. The run proceeds with the highest figure of merit for the rest of the *ARCIMBOLDO* process. In this way, it is possible to choose among alternative fragments (*i.e.* helices with different degrees of curving or helices with side chains in different conformations or fragments cut out from different homologues). Comparing the results of this approach with that starting from main-chain helices, the main difference is that the structure is solved twice after two fragments, rather than requiring the placement of three helices to obtain the first solutions. Two of the ten two-fragment solutions expanded through density modification led to recognizable solutions, with traces of 103 and 79 amino acids characterized by CCs of 26.5 and 16.1%, respectively. Their MPEs compared with the final structure were 58 and 72°, respectively. Figs. 3 ▶(*a*) and 3 ▶(*b*) display the overall structure with the located fragments superimposed and the final map and detail of the fragment placed on the final structure. As searching for successive fragments is much more time-consuming than performing many single-fragment rotations, it may be more effective to invest time initially to screen through fragments with side chains in all possible standard conformer combinations that will not clash than to have to place more fragments. Unfortunately, solving fragments may not always be unequivocally identified through such early-stage figures of merit but, in any case, it may be useful to prioritize the trials to be run.

In the case of the structure of viscotoxin A1 in space group *P*4_3_2_1_2 the asymmetric unit contains two copies of the molecule, totalling 88 amino acids. Each molecule contains two α-­helices: one of nine and another of 13 amino acids. In these cases, it is convenient to search first for two copies of the larger helix of 13 residues and then for two copies of the shorter one. From the secondary-structure prediction, the position within the helix where a cysteine is located is predetermined. Cysteines within a helix possess only two favourable conformers. Thus, this information can be exploited in the fragment. Indeed, searching for polyalanine helices gives many more solutions to the rotation function under the same conditions (94 *versus* eight) and the whole process is accelerated by searching for a helix with a cysteine side chain. What is remarkable in this case is that even solutions where the cysteine has been misplaced may lead to phasing the structure, as can be seen in Fig. 3 ▶. Figs. 3 ▶(*c*) and 3 ▶(*d*) show the final phased map, with data extrapolated to a resolution of 1.0 Å (Usón *et al.*, 2007 ▶), and misplaced helices that nevertheless led to this solution.

### 3.4. Control parameters

The Condor grid is used to allow the calculation of a large number of processes in parallel. As figures of merit cannot reliably characterize the successful solutions at their early stages, it is important to push a very large number of hypotheses to make structure solution possible. Still, it is obvious that any system will have a limit and exponentially increasing the number of jobs from fragment to fragment would swiftly lead to a collapse in the procedure. The structures provided as tutorials and used to illustrate the examples in this paper are comparatively small, but the case of eIF5 (PDB entry 2iu1; Bieniossek *et al.*, 2006 ▶), made up of 208 residues, belonging to space group *P*2_1_2_1_2_1_ and diffracting to a resolution of 1.7 Å, presents a different situation. This structure requires the location of five fragments for its solution and the unchecked flow of the program produces 201 solutions to expand for one fragment, 1366 for the second, 7465 for the third and 33 140 for the fourth. During the fifth fragment the run collapses, but manually testing some of the solutions led to correct phases. Whereas 5919 parallel jobs (generated in the equivalent run with filtering) is demanding but doable, 33 000 become utterly intractable for the file system. Even if it were possible to organize the run in such a way that they are all calculated, it is more efficient to discard trials eventually leading to failure as soon as possible and to spot those solutions that are more likely to succeed and design an express way to push them forward in the process and save time by stopping computations if they lead, as expected, to success.

The general flow of the procedure is as follows.

(i) Select one among the choice of fragments in PDB format according to an FOM.
(ii) Perform fast rotation function with *Phaser*, cutting the resolution to typically 2.1–2.5 Å.
(iii) Perform fast translation function with *Phaser* using the resolution cutoff to leave out poor data.
(iv) Perform packing filtering with *Phaser*.
(v) Perform LLG rescoring with *Phaser* to sort solutions and discard the bottom 20% of each packet.
(vi) Perform refinement and phasing with *Phaser* to improve and cluster out equivalent solutions and discard the bottom 20% of each packet.
(vii) Sort solutions according to an FOM of choice (LLG, *Z* score, number of solutions…).
(viii) Expand the top solutions through density modification and autotracing with *SHELXE* using the full resolution of the data or enhancing it through data extrapolation. If the CC exceeds a predetermined value, flag the solution and stop the process. The remaining partial structures are compressed and kept aside for further calculation if the express route fails.

This process is iterated over the number of fragments specified in the search parameters. All surviving solutions of *n* parameters are used to start possible *n* + 1 fragment structures.

The parameters that have been introduced to this end are a limit on the number of rotations to be launched, $rot_limit, and a secondary limit on the rotations, $rot_sec_limit. The first number will allow as many solutions to be taken from the resulting solution files of the last rigid-body refinement. Once this number has been filled, the second limit is used to take as many solutions from each further rigid-body packet file. The reason for this choice is that solutions are not truly independent. Within the same file, solutions tend to share some parentage. If the top figures of merit are apparent from the start, forcing a sort from the beginning would help. Otherwise, it will just make sampling more uniform. Thus, even if FOMs are lower, it is good to retain part of the various packages generated.

For the translations some optional limits can also be switched on. Solutions containing a lot of peaks within 75% of the top peak can sometimes be discarded *versus* solutions containing few peaks. This limit is relative; thus, an average of the number of solutions is estimated as from the second fragment and from that point on translation solutions exceeding this limit are completely discarded. This limit is expected to decrease from fragment to fragment. It does so in the above-illustrated case of 2iu1, but general statistics cannot be provided as they would require too much CPU time.

In addition, identifying solutions early on may be exploited to stop the whole *ARCIMBOLDO* run and avoid spending any more time on an already solved structure. To this end, an ‘express lane’ has been implemented to allow more likely to succeed partial solutions to be given priority in order to save time.

### 3.5. Configuration GUI

**Figure 4:**
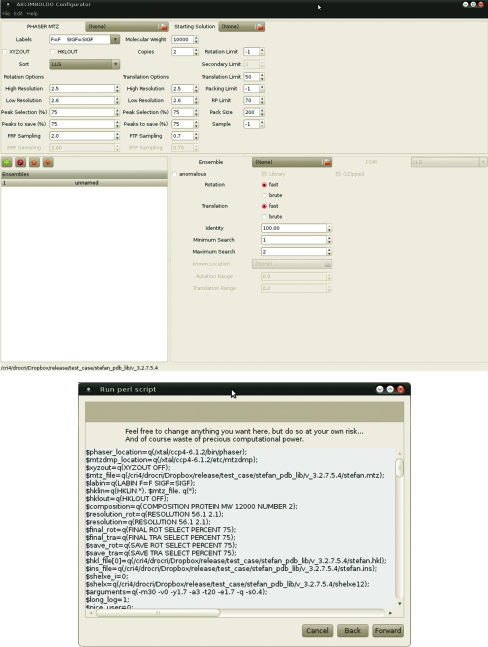
Windows of the *ARCIMBOLDO* configuration GUI.

Inputting the right choice of parameters into *ARCIMBOLDO* is tedious and error-prone. Therefore, a GUI has been programmed in C# and is distributed with the release. It allows the input of templates for different scenarios for the modification of parameters to suit the case in question. Environmental variables or paths to the executable may be changed to suit the computer system. It checks and analyzes the input files as well as the choice of parameters and will give warnings whenever any parameter or combination appears to be inappropriate. This is still not unavoidable as it may be run remotely from the site where *ARCIMBOLDO* will be run; for instance, if a run should be performed outside a graphical environment, such as a supercomputer. However, the user is allowed to override all limits. Fig. 4 ▶ shows the appearance of the GUI.

## 4. Conclusion

Brute-force multisolution combination of model fragments or alternative models consistent with previous stereochemical information, anomalous fragments or substructures and density modification and autotracing within the program *SHELXE* can be accomplished on a Condor computer grid to phase difficult macromolecular structures within the frame of *ARCIMBOLDO*.
